# Integrated management of atrial fibrillation with digital health support: a pooled analysis of individual participant data from the mAFA-II and MIRACLE-AF trials

**DOI:** 10.1093/cvr/cvag173

**Published:** 2026-08-30

**Authors:** Gregory Y H Lip, Yang Chen, Mingfang Li, Yutao Guo, Minglong Chen

**Affiliations:** Institute of Life Course and Medical Sciences, University of Liverpool, William Henry Duncan Building, 6 West Derby Street, Liverpool L7 8TX, UK; Department of Clinical Medicine, Aalborg University, Selma Lagerløfs Vej 249, Gistrup, Aalborg 9260, Denmark; Department of Cardiology, Lipidology and Internal Medicine with Intensive Coronary Care Unit, Medical University of Bialystok, 1 Kilińskiego St, Bialystok 15-089, Poland; Institute of Life Course and Medical Sciences, University of Liverpool, William Henry Duncan Building, 6 West Derby Street, Liverpool L7 8TX, UK; Department of Cardiovascular and Metabolic Medicine, Institute of Life Course and Medical Sciences, University of Liverpool, Liverpool, UK; Division of Cardiology, The First Affiliated Hospital of Nanjing Medical University, Nanjing, China; Institute of Life Course and Medical Sciences, University of Liverpool, William Henry Duncan Building, 6 West Derby Street, Liverpool L7 8TX, UK; Department of Pulmonary Vessel and Thrombotic Disease, Sixth Medical Centre, Chinese PLA General Hospital, Beijing, China; Division of Cardiology, The First Affiliated Hospital of Nanjing Medical University, Nanjing, China

**Keywords:** Atrial fibrillation, ABC pathway, Holistic management, Integrated care, Randomized trials

## Abstract

**Aims:**

Adherence with a holistic or integrated care management of atrial fibrillation (AF) based on the AF better care (ABC) pathway has been associated with improved clinical outcomes. Two prospective randomized trials (mAFA and MIRACLE-AF) have evaluated this approach. The multicentre mAFA-II trial delivered the ABC pathway via mobile health using an mAFA App, while the MIRACLE-AF trial relied on village doctors supported by telehealth in rural communities. We conducted a pooled analysis of individual participant data to assess the overall efficacy of ABC pathway-based management in patients with AF.

**Methods and results:**

We combined patient-level data from the mAFA-II and MIRACLE-AF trials. The *primary endpoint* was defined as a composite of all-cause mortality, ischaemic stroke, haemorrhagic stroke, heart failure (HF), acute coronary syndrome (ACS), and major bleeding events. The *secondary endpoints* included individual components and two grouped outcomes: all stroke and HF/ACS. A one-stage marginal Cox proportional hazards model stratified by trial and with robust standard errors clustered at the site level was used, with adjustment for CHA_2_DS_2_-VASc and other clinically relevant baseline variables. Cumulative event rates were estimated using Kaplan–Meier methods. Subgroup and sensitivity analyses were conducted to assess the robustness of the findings. Between-trial heterogeneity was further explored using a two-stage approach, incorporating trial-level effect estimates and inverse-variance weighting. We studied 4363 patients with AF [mean age 70.3 (SD 12.8) years; 60.5% male]. During 0.8 [SD 0.4] years of follow-up, the primary endpoint occurred in 329 (7.5%). Kaplan-Meier curves demonstrated lower cumulative incidence of events in the intervention group. On multivariable mixed-effects Cox models, the intervention group demonstrated a significantly lower risk of the primary endpoint compared to the control group (adjusted hazard ratio: 0.72; 95% confidence interval: 0.56–0.93). Subgroup analyses suggested potential effect modification, whereas sensitivity analyses consistently supported the primary findings. A two-stage analysis showed directionally consistent effects across trials for the primary endpoint.

**Conclusion:**

In this pooled individual participant data from two prospective randomized trials, ABC pathway-based integrated care was associated with improved clinical outcomes in patients with AF vs. usual care, with the observed benefit primarily driven by reductions in HF/ACS-related events rather than classical AF-specific outcomes, supporting further implementation studies and context-specific adoption in clinical practice.

## Graphical Abstract

**Graphical Abstract:**
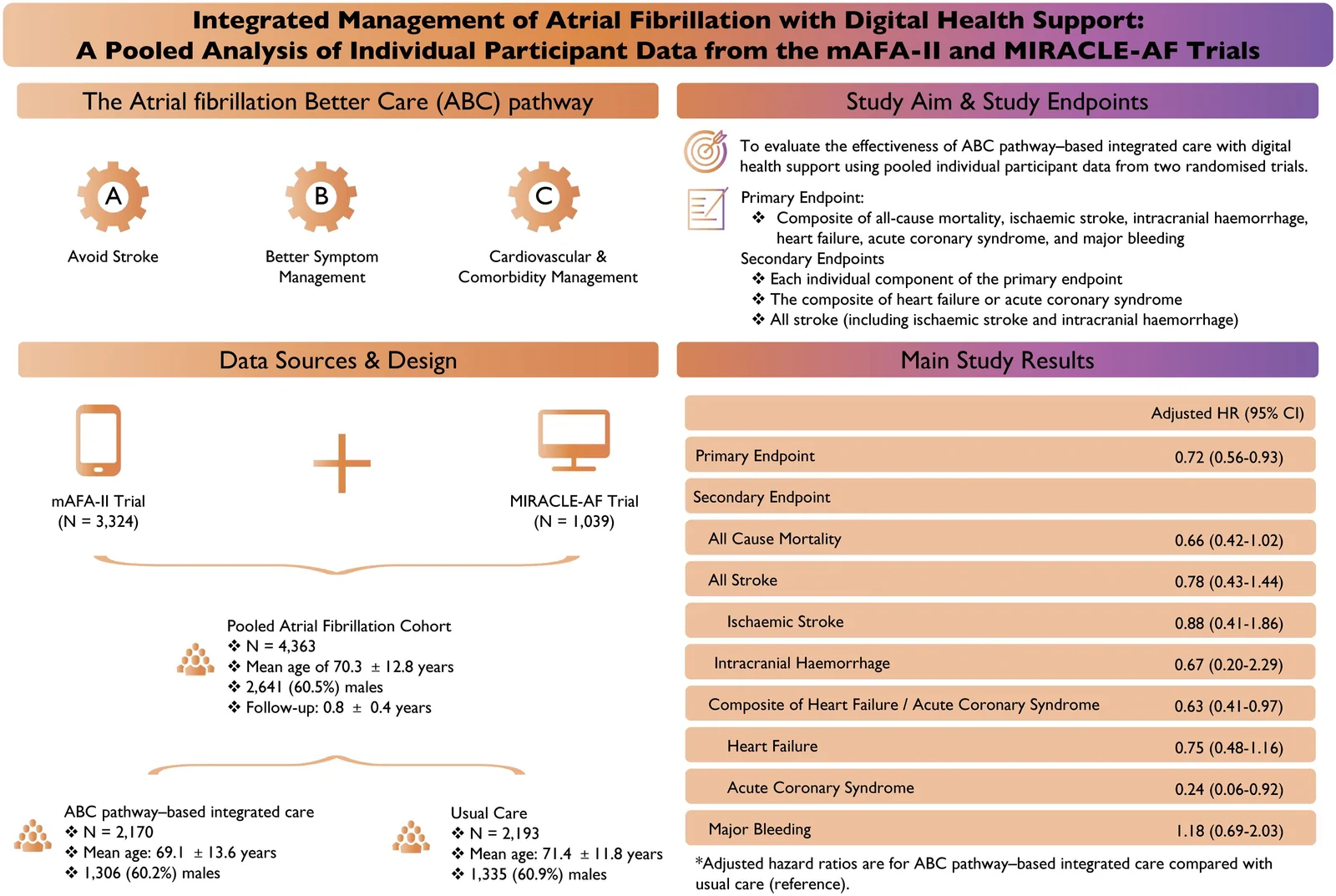

**Time of primary review: 52 days**

## 1. Introduction

Atrial fibrillation (AF) confers a high risk of mortality and morbidity from stroke and thromboembolism, heart failure, dementia, and recurrent hospitalizations, leading to a major healthcare cost burden.^1,2^ Given the increasingly elderly general population, the prevalence and incidence of AF are also increasing.^1^ There is also the recognition that AF management goes beyond anticoagulation alone, as despite the use of oral anticoagulation, a major residual risk of stroke and other major adverse cardiovascular events and hospitalizations remain.^3–6^

Hence, contemporary management of AF has moved towards a more holistic or integrated care approach, which is now promoted by major guidelines globally.^7–9^ In older small studies, integrated care management of AF was associated with improved outcomes, although there was some heterogeneity in the components.^10^ Given that AF is so common and managed in primary care and secondary settings, including emergency physicians, stroke medicine, post-operatively, and cardiologists, a simplified framework for this holistic approach was needed to facilitate uptake and implementation. In 2017, such an approach was formulated into the AF better care (ABC ) pathway, with the following components: A/avoid stroke with anticoagulation; B/better symptom and rhythm management with patient-centred symptom-directed rate or rhythm control; and C/cardiovascular risk factor and comorbidity optimization, including attention to lifestyle changes, psychological morbidity, and patient values and preferences.^11^

Adherence with a holistic or integrated care management of AF based on the ABC pathway has been tested in two prospective randomized controlled trials (RCTs), the Mobile Health Technology for Improved Screening and Optimized Integrated Care in AF (mAFA-II) trial and MIRACLE-AF (a novel Model of IntegRAted Care of oLdEr patients with Atrial Fibrillation in rural China) trial, which have evaluated this management approach.^12–15^ The multicentre mAFA-II trial delivered the ABC pathway via mobile health (mHealth) using an mAFA App largely in urban settings,^12,13^ while the MIRACLE-AF trial intervention was by village doctors in rural communities, with face-to-face delivery supported by telehealth.^14,15^ Both trials demonstrated that the ABC pathway intervention, irrespective of trial setting, was associated with improved clinical outcomes and a significant reduction in the primary composite outcome, compared to usual care.

In this article, we conducted a pooled analysis of individual participant data from the mAFA-II and MIRACLE-AF trials to assess the overall efficacy of ABC pathway-based management in patients with AF.

## 2. Methods

### 2.1 Data source

This pooled analysis included individual-level data from two cluster-randomized clinical trials: the mAFA-II trial and the MIRACLE-AF trial. The mAFA-II trial (ChiCTR-OOC-17014138) was a prospective, two-arm, cluster-randomized study enrolling AF patients in 40 participating hospitals across China. The MIRACLE-AF trial (NCT04622514) was an investigator-initiated, open-label, cluster-randomized trial conducted in 30 village clinics across Jiangdu County, Yangzhou, China. Trial protocols, study designs, and primary outcomes for both trials have been published.^12–15^ Information on participant flow, loss to follow-up, and intervention fidelity (including adherence and persistence) was obtained from the original trial reports and is summarized in Supplementary material online, *Table S1*.

Ethical approval was obtained from the Central Medical Ethics Committee of the Chinese PLA General Hospital (approval number: S2017-105-02) and local institutional review boards for the mAFA-II trial, and from the Institutional Review Board of the First Affiliated Hospital of Nanjing Medical University (approval number: 2020-SR-027) for the MIRACLE-AF trial. Both studies complied with the Declaration of Helsinki. All participants provided written informed consent before enrolment. The present pooled analysis used de-identified data and was therefore exempt from further ethics review. It was not prospectively registered, as this was a secondary analysis of existing trial datasets.

Inclusion and exclusion criteria for the mAFA-II and MIRACLE-AF trials are summarized in Supplementary material online, *Table S2*. Briefly, the mAFA-II trial enrolled patients with confirmed AF and a CHA_2_DS_2_-VASc score ≥2, recruited primarily from hospital settings. Key exclusion criteria included the presence of mechanical heart valves or moderate-to-severe mitral stenosis, inability to provide informed consent, or anticipated inability to complete 1-year follow-up. The MIRACLE-AF trial enrolled patients with confirmed AF residing in rural communities who agreed to be managed by village doctors. Exclusion criteria included prior catheter ablation or left atrial appendage occlusion, recent cardiac surgery, planned interventional procedures, rheumatic mitral stenosis, implanted cardiac devices, life-limiting conditions, or concurrent participation in other clinical studies.

### 2.2 Randomization and intervention

Both trials adopted a cluster-randomized design with 1:1 allocation. In the mAFA-II trial, participating hospitals were randomly assigned to either the intervention or usual care arm using a computer-generated random sequence. In the MIRACLE-AF trial, village clinics were similarly randomized, stratified by geographic location and clinic capacity. In both studies, randomization occurred at the cluster level.

Both trials implemented integrated AF care via digital health tools, following the ABC pathway. In the mAFA-II trial, the intervention group used a smartphone application, developed for both clinicians and patients, to facilitate ABC pathway-based management through clinical decision support tools, patient self-management, and structured follow-up. In the MIRACLE-AF trial, the intervention involved a telemedicine-based care model delivering ABC pathway management, supported by a digital platform designed to support village doctors with specialist input from tertiary hospitals. The detailed definitions of ABC pathway and implementation details of the ABC pathway via these digital platforms can be found in the previously published trial protocols, rationale papers.^12,14^ Participants in the control group received usual care in accordance with local clinical practice.

Within the ABC pathway, the ‘B’ component (Better symptom management) was delivered through structured, symptom-guided management supported by digital tools. In mAFA-II, this was achieved via app-based rhythm monitoring using photoplethysmography-enabled devices, with symptoms assessed using the European Heart Rhythm Association (EHRA) classification and communicated to clinicians to enable timely treatment adjustment. In contrast, in MIRACLE-AF, the ‘B’ component was operationalized as an outcome-based assessment of symptom control using the EHRA score at follow-up to guide patient-centred rate or rhythm control decisions. The ‘A’ and ‘C’ components were implemented in a broadly similar manner across both trials.

### 2.3 Study endpoints and follow-up

The primary endpoint of this pooled analysis was a composite of all-cause mortality, ischaemic stroke (IS), intracranial haemorrhage (ICH), heart failure (HF), acute coronary syndrome (ACS), and major bleeding. Secondary endpoints included each component of the primary endpoint, as well as a composite of HF or ACS and all stroke (including both IS and ICH). All clinical events were adjudicated by independent clinical event committees specific to each trial, blinded to treatment allocation.

The original trials used different primary endpoint definitions. In mAFA-II, the primary outcome was a composite of IS or thromboembolism, all-cause mortality, and rehospitalization, whereas MIRACLE-AF used a composite including cardiovascular death, stroke, hospitalization for worsening HF or ACS, and AF-related emergency visits.

HF and ACS events were harmonized across trials, corresponding to clinically recorded HF/ACS events in mAFA-II and hospitalization-based events in MIRACLE-AF. Given differences in endpoint definitions and ascertainment between the two trials, a harmonized composite endpoint was used to ensure comparability.

Time-to-event was defined as the duration from randomization to the first event or censoring, whichever came first. Only the first occurrence of each endpoint was considered in the primary analysis, and recurrent events were not modelled. In the MIRACLE-AF cohort, participants with incomplete follow-up were censored at the last available follow-up time within the time-to-event framework. To minimize potential bias arising from differing follow-up durations between the two trials (maximum follow-up: 439 days in mAFA-II vs. 36 months in MIRACLE-AF), the MIRACLE-AF dataset was truncated at 493 days to match the mAFA-II trial.

### 2.4 Statistical analyses

This pooled analysis included intention-to-treat populations. All covariates included in the analysis were complete, with no missing data requiring imputation. A one-stage approach was used to analyse individual patient data. Continuous variables were expressed as mean ± standard deviation (SD) or median (interquartile range), with group comparisons conducted using the *t* test or Mann–Whitney *U* test based on distributional assumptions. Frequencies and proportions were used to summarize categorical variables, with comparisons performed using the χ^2^ test. Event rates were calculated per person-year, and rate differences (RDs) with 95% confidence intervals (CIs) were estimated using a Poisson distribution. Kaplan–Meier curves were used to estimate and compare the cumulative incidence of the primary outcome between groups.

The primary analysis was performed using marginal Cox proportional hazards models stratified by trial and with robust sandwich standard errors clustered at the level of the original randomization unit (site), to account for both between-trial differences in baseline hazard and within-cluster correlation.^16^ Models were adjusted for the CHA_2_DS_2_-VASc score, HAS-BLED score, and clinically relevant baseline variables, showing imbalance between intervention and control groups. Adjusted hazard ratios (aHRs) and 95% CIs were reported. The proportional hazards assumption was assessed using Schoenfeld residuals (see Supplementary material online, *Table S3*). Subgroup analyses were conducted across predefined strata, including age (≥65 vs. <65 years), AF type (paroxysmal vs. non-paroxysmal AF), and CHA_2_DS_2_-VASc risk (males ≥3 or females ≥4 are considered high risk), sex, baseline anticoagulation status, hypertension, diabetes mellitus, CAD, HF, CKD, and prior IS. Trial-stratified Cox proportional hazards models with robust standard errors clustered at the site level were used, and interaction terms between treatment and subgroup variables were included to explore potential effect modification.

To evaluate the robustness of the findings, we conducted several sensitivity analyses. First, mixed-effects Cox proportional hazards models with site-level random intercepts were fitted as an alternative approach to account for within-cluster correlation. Second, to account for the competing risk of death, we applied Fine–Gray subdistribution hazard models to non-fatal secondary endpoints. Third, we repeated the main analysis for primary endpoint by truncating follow-up durations to 6 and 12 months to examine the consistency of treatment effects in the short term. Fourth, to minimize reverse causality, 1-month and 3-month landmark analyses for the primary endpoint were conducted by excluding early events and restarting follow-up from each landmark among event-free patients. Follow-up was recalibrated for each landmark point among those who were event-free at that time.

To assess the robustness of the findings to endpoint definition, analyses of the original trial-specific primary endpoints were performed within each trial population using the same modelling framework.

Additionally, a pre-specified two-stage approach (package ‘meta*’* in R) for primary and secondary endpoints (excluding ICH due to no events in the intervention group of the mAFA trial) was employed to assess the consistency of treatment effects across trials. Trial-specific aHRs, standard errors, and 95% CIs, obtained from Cox proportional hazards models adjusted for CHA_2_DS_2_-VASc score, were synthesized using an inverse-variance weighted fixed-effect approach. Inter-trial heterogeneity was evaluated through Cochran’s Q statistic and quantified using the *I^2^ metric*.

Statistical significance was determined at a two-sided *P* value <0.05, without correction for multiple testing. All analyses were conducted using R software (version 4.2.1; R Foundation for Statistical Computing, Vienna, Austria).

## 3. Results

### 3.1 Baseline characteristics

A total of 4363 patients [mean (SD) age: 70.3 (12.8) years; 2641 (60.5%) males] were included in this pooled analysis, with 2193 in the control group [mean (SD) age: 71.4 (11.8) years; 1335 (60.9%) males] and 2170 in the intervention group [mean (SD) age: 69.1 (13.6) years; 1306 (60.2%) males]. The intervention group was younger on average, while CHA_2_DS_2_-VASc scores were comparable between groups. Most comorbidities were similarly distributed. Detailed baseline characteristics, including medication use, are summarised in *Table 1*.

**Table 1:** Baseline characteristics

Characteristics	Control	Intervention	*P* value
N	2193	2170	
Age, years	71.4 ± 11.8	69.1 ± 13.6	<0.001
Male, *n* (%)	1335 (60.9)	1306 (60.2)	0.663
Current smoker, *n* (%)	290 (13.2)	254 (11.7)	0.141
Paroxysmal AF, *n* (%)	700 (31.9)	710 (32.7)	0.595
CHA_2_DS_2_-VASc score	2.9 ± 1.5	3.0 ± 1.6	0.153
HAS-BLED score	1.6 ± 1.0	1.5 ± 1.1	0.002
Comorbidities/clinical history, *n* (%)			
Hypertension	1406 (64.1)	1354 (62.4)	0.252
Diabetes mellitus	467 (21.3)	473 (21.8)	0.714
Chronic kidney disease	267 (12.2)	221 (10.2)	0.042
Heart failure	388 (17.7)	387 (17.8)	0.934
Coronary artery disease	734 (33.5)	646 (29.8)	0.009
Peripheral artery disease	189 (8.6)	191 (8.8)	0.872
Liver dysfunction	102 (4.7)	131 (6.0)	0.049
Dilated cardiomyopathy	76 (3.5)	55 (2.5)	0.087
Hypertrophic cardiomyopathy	31 (1.4)	28 (1.3)	0.825
Prior ischaemic stroke	305 (13.9)	277 (12.8)	0.287
Prior systemic embolism	59 (2.7)	56 (2.6)	0.895
Prior intracranial bleeding	39 (1.8)	25 (1.2)	0.111
Prior other major bleeding	68 (3.1)	55 (2.5)	0.299
Prior catheter ablation	173 (7.9)	184 (8.5)	0.512
Prior pacemaker implantation	87 (4.0)	81 (3.7)	0.746
Medications, n (%)			
Warfarin	194 (8.8)	100 (4.6)	<0.001
Dabigatran	416 (19.0)	805 (37.1)	<0.001
Rivaroxaban	256 (11.7)	240 (11.1)	0.555
Aspirin	388 (17.7)	249 (11.5)	<0.001
Clopidogrel	133 (6.1)	89 (4.1)	0.004
Propafenone	52 (2.4)	33 (1.5)	0.055
Amiodarone	82 (3.7)	200 (9.2)	<0.001
Beta-blockers	417 (19.0)	612 (28.2)	<0.001
Digoxin	164 (7.5)	178 (8.2)	0.404
ACEIs/ARBs	532 (24.3)	580 (26.7)	0.066
Calcium antagonists	357 (16.3)	414 (19.1)	0.017
Diuretics	423 (19.3)	310 (14.3)	<0.001
Statins	607 (27.7)	695 (32.0)	0.002
Nitrates	284 (13.0)	229 (10.6)	0.016
Gastric antiacid drugs	220 (10.0)	139 (6.4)	<0.001

Values are presented as mean ± SD for continuous variables, *n* (%) for categorical variables. *P* values are based on Student’s *t*-test for continuous variables and χ^2^ test for categorical variables.

AF, atrial fibrillation; ACEIs, angiotensin-converting enzyme inhibitors; ARBs, angiotensin receptor blockers.

### 3.2 Clinical outcomes and treatment effect estimates

During a mean follow-up of 0.8 ± 0.4 years, a total of 159 (7.3%) primary endpoints occurred in the control group and 100 (4.6%) in the intervention group. *Table 2* shows the absolute event rates for the primary and secondary endpoints. For the primary endpoint, the event rate was lower in the intervention group (5.7% per year) than in the control group (8.5% per year), with an RD of −2.81% (95% CI: −4.54 to −1.09; *P* = 0.001), corresponding to an absolute risk reduction of 2.8% and an approximate number needed to treat (NNT) of 36.

**Table 2:** Clinical outcomes in the intervention and control groups

Study endpoint	Intervention		Control		Rate Difference (95% CI) (%)^a^	*P* value
	No. of events	Rate per year (%)	No. of events	Rate per year (%)		
Primary endpoint^b^	100	5.7	159	8.5	−2.81 (−4.54, −1.09)	0.001
Secondary endpoints						
All-cause mortality	35	2.0	59	3.1	−1.11 (−2.12, −0.10)	0.032
All stroke	13	0.7	18	0.9	−0.21 (−0.80, 0.38)	0.481
IS	10	0.6	12	0.6	−0.06 (−0.55, 0.43)	0.804
ICH	4	0.2	7	0.4	−0.14 (−0.48, 0.21)	0.428
Composite of HF/ACS	35	2.0	60	3.2	−1.19 (−2.22, −0.16)	0.024
HF	34	1.9	51	2.7	−0.76 (−1.73, 0.21)	0.126
ACS	3	0.2	13	0.7	−0.51 (−0.92, −0.09)	0.016
Major bleeding	33	1.9	41	2.3	−0.31 (−1.27, 0.64)	0.522

ACS, acute coronary syndrome; CI, confidence interval; HF, heart failure; ICH, intracranial haemorrhage; IS, ischaemic stroke.

^a^Rate differences and their 95% CIs were estimated under the assumption of a Poisson distribution of events, using standard errors derived from event counts and person-years. Two-sided Z-tests were used to calculate *P* values.

^b^Defined as a composite of all-cause mortality, all stroke, HF, ACS, and major bleeding.

The Kaplan–Meier curves for the primary endpoint are shown in *Figure 1*. Based on trial-stratified Cox proportional hazards models with robust standard errors clustered at the site level (*Figure 2*), the primary endpoint occurred less frequently in the intervention group compared with the control group, corresponding to an aHR of 0.72 (95% CI: 0.56–0.93; *P* = 0.011). Among secondary endpoints, significant reductions were observed in the composite of HF or ACS, while estimates for ACS alone were based on a small number of events.

**Figure 1:**
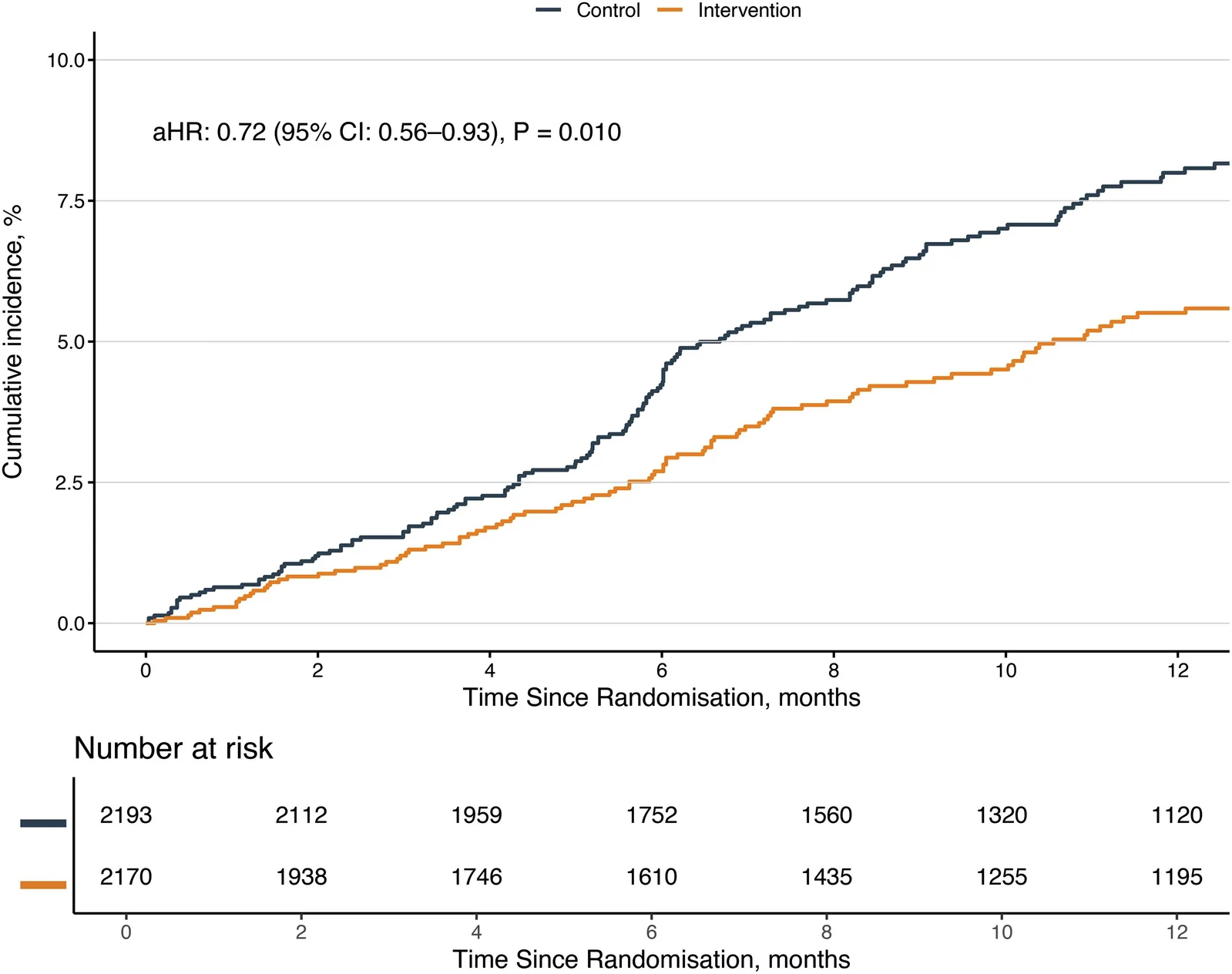
Kaplan–Meier curves for the primary endpoint. The aHRs and 95% CIs were estimated using marginal Cox proportional hazards models stratified by trial, with robust sandwich standard errors clustered at the site level. Models were adjusted for CHA_2_DS_2_-VASc score, HAS-BLED score, age, chronic kidney disease, coronary artery disease, liver dysfunction, and baseline medications (warfarin, dabigatran, aspirin, amiodarone, beta-blockers, diuretics, and gastric antiacid drugs). aHR, adjusted hazard ratio; CI, confidence interval.

**Figure 2:**
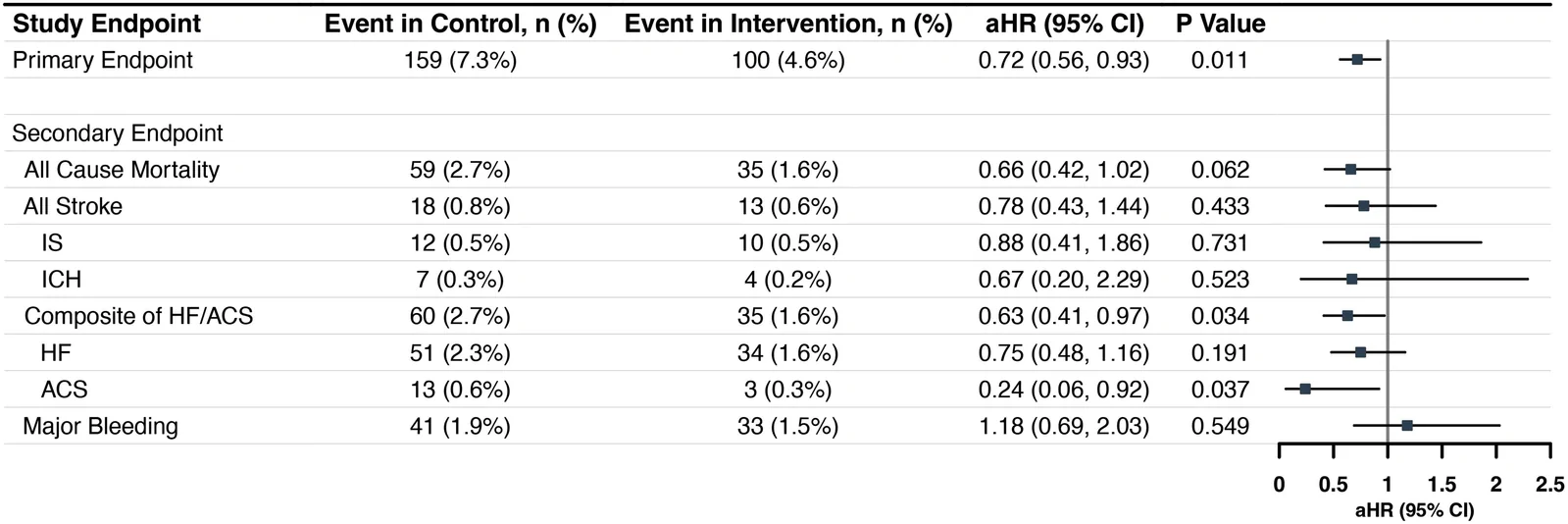
Marginal Cox proportional hazards models for primary and secondary endpoints. The aHRs and 95% CIs were estimated using marginal Cox proportional hazards models stratified by trial, with robust sandwich standard errors clustered at the site level. Models were adjusted for the CHA_2_DS_2_-VASc score, HAS-BLED score, and clinically relevant baseline variables, showing imbalance between intervention and control groups. The model compared the intervention with the control group (usual care) for each endpoint. ACS, acute coronary syndrome; aHR, adjusted hazard ratio; CI, confidence interval; HF, heart failure; IS, ischaemic stroke; ICH, intracranial haemorrhage.

### 3.3 Subgroup analyses

In subgroup analyses, several statistically significant interactions were observed for selected variables, including sex, CHA_2_DS_2_-VASc score, hypertension, CAD, HF, baseline anticoagulant use, and statin use (see Supplementary material online, *Table S4*).

### 3.4 Sensitivity analyses

Results were broadly consistent across sensitivity analyses, including mixed-effects Cox models with site-level random intercepts, Fine–Gray competing risk models, truncated follow-up at 6 and 12 months, and 1- and 3-month landmark analyses (see Supplementary material online, *Tables S5*–*S8*).

### 3.5 Analyses using trial-specific endpoints

Analyses using the original trial-specific primary endpoints within each trial population yielded consistent and numerically stronger effect estimates compared with the harmonized composite endpoint used in this pooled analysis (see Supplementary material online, *Table S9*).

### 3.6 Two-stage analysis

A two-stage approach was conducted to evaluate the consistency of treatment effects across trials (*Figure 3*). For the primary endpoint, heterogeneity estimates were low (*I*^2^ = 0.2%; Cochran’s *Q* = 1.19; *P* = 0.276), with directionally consistent effects across trials. No strong heterogeneity was observed across secondary endpoints.

**Figure 3:**
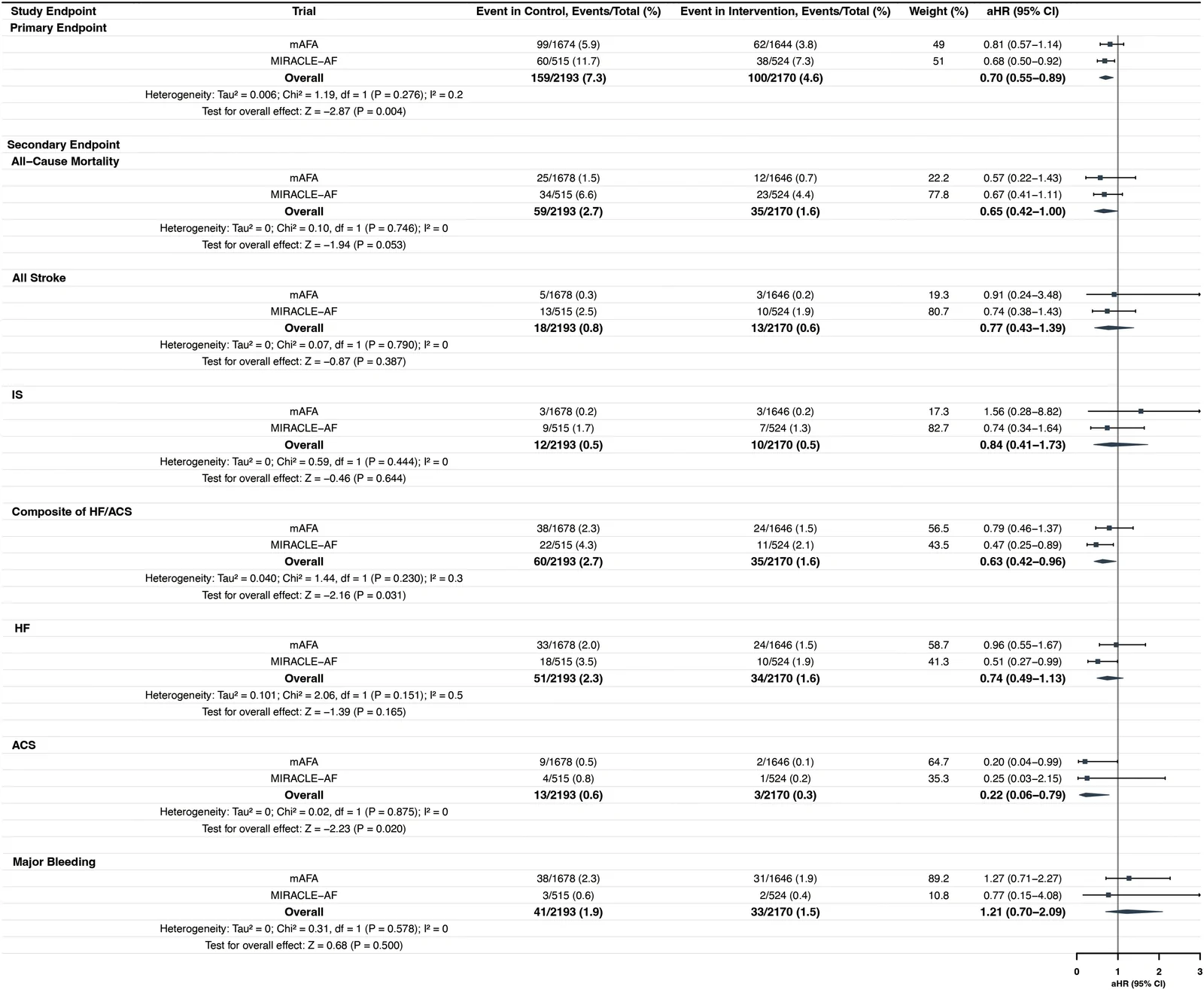
Two-stage meta-analysis of trial-level treatment effects. Trial-specific aHRs and 95% CIs were obtained from Cox proportional hazards models adjusted for CHA_2_DS_2_-VASc score, HAS-BLED score, age, chronic kidney disease, coronary artery disease, liver dysfunction, and baseline medications (warfarin, dabigatran, aspirin, amiodarone, beta-blockers, diuretics, and gastric antiacid drugs), and pooled using inverse-variance weighting under a fixed-effect model. ACS, acute coronary syndrome; aHR, adjusted hazard ratio; CI, confidence interval; HF, heart failure; IS, ischaemic stroke; ICH, intracranial haemorrhage.

## 4. Discussion

In this pooled individual participant data from two prospective RCTs, our principal findings are as follows: (i) integrated care based on the ABC pathway was associated with improved clinical outcomes in patients with AF compared with usual care, primarily driven by reductions in HF/ACS-related events, and therefore should not be interpreted as reflecting a uniform effect across all components of the composite endpoint and (ii) these findings support further implementation studies and context-specific adoption of the ABC pathway in clinical practice. No significant heterogeneity was observed between studies, with both trials showing directionally consistent effects favouring the ABC pathway intervention.

This pooled analysis supports the evidence-based ABC pathway intervention to manage AF patients in a holistic or integrated care manner. Various cohort studies, including retrospective cohorts, prospective registries, and post hoc analyses of randomized trials (hence, adjudicated outcomes), have shown that ABC pathway adherence was associated with a significant reduction in all-cause and cardiovascular mortality, as well as a reduction in stroke and major bleeding.^17,18^ Consistent with these findings, the estimated NNT of approximately 36 suggests a clinically meaningful absolute benefit of ABC pathway–based integrated care. Although NNTs are not directly comparable across studies because of differences in study populations and endpoint definitions, this estimate further supports the clinical relevance of integrated AF care. However, in the present analysis, the association with all-cause mortality should be interpreted with caution, as it did not reach statistical significance after full adjustment and may reflect the combined influence of multiple care processes rather than a direct effect. Nevertheless, in real-world settings, ABC pathway adherence was generally low and may change over time.^19^ In the COOL-AF registry, patients with AF who were ABC pathway compliant at baseline and follow-up had the best clinical outcomes, compared to those who were non-compliant at baseline and/or follow-up points.^19,20^ These findings align with recent evidence from systematic reviews and meta-analyses.^21^ Hence, the importance of ensuring adherence and persistence of such a holistic intervention approach. In the long-term extension of the mAFA-II trial, adherence (>70%) and persistence of use (>90%) remained high, notwithstanding that this trial was based on the ABC pathway delivered by a mHealth-based mAFA app.^22^

In the mAFA-II trial, 1646 patients were allocated to the ABC pathway intervention, which showed lower rates of the composite outcome of ‘IS/systemic thromboembolism, death, and rehospitalization’ compared with usual care [1.9% vs. 6.0%; hazard ratio (HR): 0.39; 95% CI: 0.22–0.67; *P* < 0.001].^12,13^ Of the secondary outcomes, rehospitalization rates were lower with the ABC pathway intervention (HR: 0.32; 95% CI: 0.17–0.60; *P* < 0.001). Additional subgroup analyses from the mAFA-II trial clearly show the benefits on the primary composite outcome were consistent in the elderly, secondary prevention, HF, and diabetes, irrespective of sex.^23–26^ The ABC pathway intervention has also been shown to be cost effective.^27^ Indeed, a modelling health economic exercise clearly showed that adherence to the ABC pathway was projected to reduce mortality, strokes, and bleedings over the next decades, leading to lowered healthcare costs.^28^

The MIRACLE-AF trial was performed in a very different setting using a telemedicine-based, village doctor-led integrated care model to deliver the ABC pathway intervention, compared to enhanced usual care.^14,15^ In this trial, the rate of the composite cardiovascular event outcome was reduced in the intervention cluster than in the usual care group (6.2% vs. 9.6% per year; HR: 0.64; 95% CI: 0.50–0.82; *P* < 0.001). In MIRACLE-AF, the pathway was delivered by village doctors, many of whom were elderly and not medically qualified.^29^ Various ancillary analyses from MIRACLE-AF are in progress, including those demonstrating the beneficial effects in high-risk subgroups and their cost-effectiveness (data not shown).

These data are supplemented by numerous real-world observational cohort studies, from different healthcare settings, national studies and global registries, and different parts of the world bleeding.^17,18^ These findings align with prior evidence from structured care models. In a randomized trial by Hendriks *et al.*, nurse-led integrated care reduced cardiovascular hospitalization or death (HR: 0.65; 95% CI: 0.45–0.93), supporting the value of multidisciplinary, protocol-based management.^30^ Unlike the digital health–supported interventions evaluated in the present study, the Hendriks *et al*. trial employed a nurse-led integrated care model delivered through specialized AF clinics. Given that patients with AF are not a homogeneous group, with risk profiles dependent on associated comorbidities that tend to cluster, the beneficial impact of the ABC pathway was observed in clinically complex phenotypes, irrespective of risk profile–based clinical clusters.^31–34^

The absence of a statistically significant reduction in stroke outcomes should be interpreted in context. In the present analysis, IS events were infrequent (12 in the intervention group and 10 in the control group), limiting statistical power for stroke-specific comparisons. Notably, baseline anticoagulation use was higher in the intervention group (52.7%) than in the control group (39.0%), which may have attenuated the incremental benefit of the intervention. Despite this, the direction of effect for stroke remained consistent with the overall findings (adjusted hazard ratio <1), suggesting a potential benefit that may not have been detectable within the current sample size and follow-up duration. Similarly, the estimate for ACS should be interpreted in the context of a small number of events, which may limit the precision of the effect estimate.

The observed reduction in the composite HF/ACS outcome is nevertheless biologically plausible within the framework of the ABC pathway. Beyond stroke prevention, integrated care emphasizes optimization of cardiovascular risk factors, evidence-based management of comorbidities, medication adherence, and regular clinical review, all of which may reduce the risk of cardiovascular decompensation and acute cardiovascular events. Therefore, although the estimate for ACS alone should be interpreted with caution given the limited number of events, the overall reduction in HF/ACS-related outcomes is consistent with the broader cardiovascular benefits expected from integrated care than with AF-specific mechanisms alone.

The role of the ‘B’ (Better symptom management) component warrants consideration. In both trials, symptom-guided care was delivered via digital pathways, with app-based monitoring and dynamic adjustment in mAFA-II and follow-up–based, clinician-led management in MIRACLE-AF. Although ‘B’-related data were available in each trial (EHRA-based assessment in MIRACLE-AF; monitoring adherence in mAFA-II extension analyses),^15,22^ these measures differed in definition and timing and were not harmonized. Consequently, the contribution of the ‘B’ component to patient-centred outcomes cannot be directly quantified in this pooled analysis.

The handling of hospitalization warrants consideration when interpreting these findings. Although rehospitalization was a key component of the primary endpoint in mAFA-II, it was not included in the harmonized composite due to differences in definition and ascertainment across trials, with broader capture in mAFA-II and more specific hospitalization-based events in MIRACLE-AF. Including these heterogeneous definitions would have reduced comparability and introduced potential bias. The use of a harmonized composite endpoint in this pooled analysis reflects differences in trial-specific definitions and was intended to enable comparability across trials, rather than to redefine trial-specific outcomes. Importantly, analyses based on the original trial-specific endpoints yielded consistent and numerically stronger effect estimates, supporting the robustness of our findings. However, the exclusion of hospitalization may underestimate the full patient-centred and healthcare utilization benefits of the ABC pathway, and future studies with prospectively harmonized endpoints, including patient-centred outcomes such as hospitalization, are warranted.

Subgroup analyses provide additional context. Although some patterns were observed across clinically relevant strata, several statistically significant interactions were identified. These findings were not consistent across related variables and may reflect chance findings arising from multiple comparisons. Subgroup analyses were exploratory in nature and not adjusted for multiple testing. Therefore, these results should be interpreted with caution and do not support treatment stratification; rather, they serve to generate hypotheses for future studies.

Building on these findings, this analysis also provides methodological insights relevant to future studies. The use of cluster-randomized, pragmatic trial designs enabled evaluation of integrated care strategies in real-world settings, while digital-supported delivery facilitated implementation across different healthcare contexts. However, differences in endpoint definitions highlight the importance of prospectively harmonized outcomes to improve comparability across studies. Future trials should also incorporate component-level adherence measures and patient-reported outcomes to better capture patient-centred effects and to disentangle the contributions of individual elements of the ABC pathway.

The ABC pathway has since been promoted in various guidelines globally, given its evidence-base.^35,36^ Variants of the acronym (untested in clinical trials) have been used in the 2023 ACC/AHA/ACCP/HRS guidelines (as ‘SOS’)^37^ and the 2024 ESC guidelines (as ‘CARE’).^38^ The latter proposed ‘CARE’ to give prominence to comorbidities, but with any cardiovascular disorder (and AF is no exception) attention to cardiovascular risk factors, comorbidities, and lifestyle changes is the initial management plan of action. In addition, the ABC pathway approach also emphases attention to psychological morbidity, given its recognized impact on patients with AF, as well as attention to patient values and preferences (which would facilitate shared decision-making).^11^ As risk is not static but dynamic,^39,40^ and adherence to holistic care management (even with the ABC pathway) is also dynamic,^19^ regular reassessment of risk and adherence/persistence with ABC pathway management is essential, and recommended as part of patient follow-up. While the present findings support the structured integrated care strategies represented by the ‘C’(Comorbidity management), ‘A’(Avoiding stroke), and ‘R’(Rate/rhythm control) components of the AF-CARE framework, further studies incorporating longitudinal evaluation and patient-reported outcomes are needed to evaluate the ‘E’ (Evaluation) component.

### 4.1 Limitations

This study has limitations. The two trials were designed as cluster randomized trials, in different healthcare settings, but consistency of treatment effects across trials was seen. A mean follow-up of 0.8 years limits the ability to assess long-term outcomes, particularly for endpoints such as stroke and mortality. Given that only two trials were included, between-study heterogeneity could not be reliably assessed, and estimates should be interpreted with caution. In addition, adjustment for baseline oral anticoagulant use may have resulted in partial over-adjustment, given that optimization of oral anticoagulation represents a core component of the ABC pathway. The exclusion of hospitalization from the harmonized composite endpoint, due to differences in definition across trials, may underestimate the full patient-centred and healthcare utilization benefits of the intervention. The lack of harmonized measures for symptom-related outcomes across trials limits the evaluation of patient-centred effects. Both trials were also conducted in China, and additional studies in different populations and ethnic groups are needed, given the reported racial differences in the clinical epidemiology of AF,^41^ as well as AF-related outcomes (e.g. stroke, bleeding)^42,43^ The Horizon Europe funded AFFIRMO trial is testing the ABC pathway in elderly multimorbid patients with AF in five European countries and will report in 2026.^44^

In conclusion, in this pooled individual-participant data from two prospective RCTs, integrated care based on the ABC pathway was associated with improved clinical outcomes in patients with AF vs. usual care, with the observed benefit primarily driven by reductions in HF/ACS-related events rather than classical AF-specific outcomes. These findings support further implementation studies and context-specific adoption of the ABC pathway in clinical practice.

## Supplementary Material

cvag173_Supplementary_Data

## Data Availability

The data used in this study are not publicly available due to institutional and ethical restrictions.

## Pre-registered clinical trial number

MAFA-II trial (ChiCTR-OOC-17014138), MIRACLE-AF trial (NCT04622514).
