# Supplementary material for: Integrated management of atrial fibrillation with digital health support: a pooled analysis of individual participant data from the mAFA-II and MIRACLE-AF trials
Source: Cardiovasc Res. 2026 Aug 30;122(14):1936–46. doi: 10.1093/cvr/cvag173 (PMC13653501; doi:10.1093/cvr/cvag173)
Supplement: Supplementary file 1 [file cvag173_supplementary_data.docx]

**MAFA II Investigators List**

**Executive Steering Committee:**

| Yutao Guo | Chinese PLA General Hospital, Beijing, China (Co-Chair) |
| --- | --- |
| Gregory Y H Lip | Institute of Cardiovascular Sciences, University of Birmingham, UK; Liverpool Centre for Cardiovascular Science, University of Liverpool, UK (Co-chair) |
| Deirdre A. Lane | Liverpool Centre for Cardiovascular Science, University of Liverpool, UK |
| Yundai Chen | Chinese PLA General Hospital, Beijing, China |
| Liming Wang | The National Center for Chronic and Noncommunicable Disease Control and Prevention, Chinese Center for Disease Control and Prevention, Beijing, China |

**Steering committee:**

| Jens Eckstein | University Hospital Basel, Switzerland |
| --- | --- |
| G Neil Thomas | Institute of Applied Health Research, University of Birmingham, United Kingdom |
| Liu Tong | The Second Hospital of Tianjin Medical University, Tianjin, China |
| Feng Mei | Shanxi Dayi Hospital, Taiyuan, Shanxi, China |
| Liu Xuejun | Affiliated First Hospital, Shanxi Medical University, China |
| Li Xiaoming | Cardiovascular Disease Hospital of Shanxi Province, China |
| Shan Zhaoliang | PLA General Hospital, Beijing, China |
| Shi Xiangming | PLA General Hospital, Beijing, China |
| Zhang Wei | PLA Army General Hospital, Beijing, China |
| Xing Yunli | Beijing Friendship Hospital, Capital Medical University, Beijing, China |
| Wen Jing | Beijing Haidian Hospital, Beijing, China |
| Wu Fan | Tianjin Medical University General Hospital, Tianjing, China |
| Yang Sitong | The First Affiliated Hospital, Ji Lin University, Ji Lin, China |
| Jin Xiaoqing | Tongji Hospital, Tongji medical College, Huazhong University Of Science & Technology, Wuhan, China |
| Yang Bo | Xiangya Hospital Central South University, Changsha, China |
| Bai Xiaojuan | ShengJing Hospital of China Medical University, Shengyang, China |
| Jiang Yuting | Suqian Hospital, Jiangsu, China |
| Liu Yangxia | General Hospital of Shengyang Military, Shengyang, China |
| Song Yingying | Bozhou Renmin Hospital, Anhui, China |
| Tan Zhongju | The First Hospital of Zhejiang Province, Hangzhou, China |
| Yang Li | Yunnan Cardiovascular Hospital, Kunming, China |
| Luan Tianzhu | The First Affiliated Hospital of Haerbing Medical University, Haerbing, China |
| Niu Chunfeng | The Second Affiliated Hospital of Haerbing Medical University, Haerbing, China |
| Zhang Lili | The Fourth Affiliated Hospital of Haerbing Medical University, Haerbing, China |
| Li Shuyan | The First Affiliated Hospital, Ji Lin University, Ji Lin, China |
| Wang Zulu | General Hospital of Shengyang Military, Shengyang, China |
| Xv Bing | The First People's Hospital of Shengyang, Shengyang, China |
| Liu Liming | The Second Affiliated Hospital of Shengyang Medical University, Shengyang, China |
| Jin Yuanzhe | The Fourth Affiliated Hospital of China Medical University, Shengyang, China |
| Xia Yunlong | The First Affiliated Hospital of Dalian Medical University, Dalian, China |
| Chen Xiaohong | The People's Hospital of Liaoning Province, Shengyang, China |
| Wu Fang | Rui Jin Hospital, Tong university School of Medicine, Shanghai, China |
| Zhong Lina | The Affiliated Hospital of Qingdao University, Qingdao, China |
| Sun Yihong | China-Japan Friendship Hospital, Beijing, China |
| Jia Shujie | Beijing Anzhen Hospital, Capital Medical University, Beijing, China |
| Li Jing | Xuanwu Hospital Capital Medical University, Beijing, China |
| Li Nan | The Third People’s Hospital of Dalian, Dalian, China |
| Li Shijun | Dalian Municipal Central Hospital Affiliated of Dalian Medical University, Dalian, China |
| Liu Huixia | Guangdong Academy of Medical Sciences Guangdong General Hospital, Guangdong, China |
| Li Rong | The First Affiliated Hospital of Guangzhou University of Traditional Chinese Medicine, Guangzhou, China |
| Liu Fan | The Second Hospital of Hebei Medical University, Hebei, China |
| Ge Qingfeng | North China University Science And Technology Affiliated Hospital |
| Guan Tianyun | The Second Hospital of Jilin University, Jilin, China |
| Wen Yuan | The Second Affiliated Hospital of Nanchang University, Nanchang, China |
| Li Xin | BenQ Hospital affiliated to Nanjing Medical University, Nanjing, China |
| Ren Yan | Ruijin Hospital, Shanghai Jiao Tong University School of Medicine |
| Chen Xiaoping | Taiyuan City Central Hospital, Taiyuan, China |
| Chen Ronghua | Tangshan People's Hospital, Tangshan, China |
| Shi Yun | Tianjin Fourth Central Hospital , Tianjin, China |
| Zhao Yulan | The Second Affiliated Hospital of Zhengzhou University, Zhengzhou, China |
| Shi Haili | Zhengzhou Central Hospital Affiliated to Zhengzhou University, Zhengzhou, China |
| Zhao Yujie | Zhengzhou Seventh People's Hospital, Zhengzhou, China |
| Wang Quanchun | Shenyang Fifth People's Hospital, Shenyang, China |
| Sun Weidong | Taian City Central Hospital, Taian, China |
| Wei Lin | Harbin First Hospital, Harbin, China |

**Data Safety Monitoring Board:**

| Esther Chan | The University of Hong Kong, Hong Kong, China |
| --- | --- |
| Shan Guangliang | Department of Epidemiology and Statistics, Institute of Basic Medical Sciences, Peking Union Medical College, Beijing, China |
| Yao Chen | Peking University Clinical Research Institute, Beijing, China |
| Zong Wei | China Foreign Affairs University, Beijing, China |
| Chen Dandi | West China School of Public Health, Chengdu, China |

**Clinical events committee:**

| Han Xiang | Department of Neurology, Huashan Hospital of Fudan University, Shanghai, China |
| --- | --- |
| Xu Anding | Department of Neurology, the First Affiliated Hospital of Jinan University, Guang Zhou, China |
| Fan Xiaohan | Fuwai Hospital, Chinese Academy of Medical Sciences, Beijing, China |
| Yu Ziqiang | Institute of Blood Research of Jiangsu Province, China |
| Gu Xiang | Department of Cardiology, People’s Hospital of Subei, Affiliated Hospital of YangZhou University, Jiangsu Province, China |
| Ge Fulin | Department of Gastroenterology, Chine PLA General Hospital, Beijing, China |

**MIRACLE-AF Investigators List**

**Steering Committee:**

| Minglong Chen | Cardiologist, The First Affiliated Hospital of Nanjing Medical University |
| --- | --- |
| Gregory Y. H. Lip | Cardiologist, Liverpool Centre for Cardiovascular Science, University of Liverpool |
| Yutao Guo | Cardiologist, Chinese PLA General Hospital |
| Xinfeng Liu | Neurologist, Jinling Hospital, Medical School of Nanjing University, Nanjing, China |
| Shanshan Lu | Radiologist, The First Affiliated Hospital of Nanjing Medical University |

**Data Safety Monitoring Board:**

| Wenxi Wu | General Surgery, The First Affiliated Hospital of Nanjing Medical University |
| --- | --- |
| Di Xu | Geriatric Cardiologist, The First Affiliated Hospital of Nanjing Medical University |
| Ping Gu | Neurologist, The First Affiliated Hospital of Nanjing Medical University |
| Tianping Li | Research Division of Clinical Pharmacology, The First Affiliated Hospital of Nanjing Medical University |
| Haipeng Xu | Statistician, The First Affiliated Hospital of Nanjing Medical University |

**Clinical events Committee:**

| Fumin Zhang | Cardiologist, The First Affiliated Hospital of Nanjing Medical University |
| --- | --- |
| Liqun Ren | Cardiologist, Zhongda Hospital, School of Medicine, Southeast University, Nanjing, Jiangsu, China |
| Wusheng Zhu | Neurologist, Jinling Hospital, Medical School of Nanjing University, Nanjing, China |
| Hong Cheng | Neurologist, The First Affiliated Hospital of Nanjing Medical University |
| Xiaoquan Xu | Radiologist, The First Affiliated Hospital of Nanjing Medical University |

**Contract research organization (CRO)**

| Item | Title | Country |
| --- | --- | --- |
| Contract Research Organization (CRO) | Guangzhou EnChannel Medical Information Technology Co. Ltd | China |

**Supplementary Table S1. Participant flow, follow-up completeness, and intervention fidelity in the included trials**

| **Domain** | **mAFA-II** | **MIRACLE-AF** |
| --- | --- | --- |
| Cluster level |  |  |
| Cluster assessed | 42 | 70 |
| Clusters randomised | 40 (20 / 20) | 30 (15 / 15) |
| Clusters excluded | 2 | 40 |
|  |  |  |
| Patient screening and enrolment |  |  |
| Patients assessed | 1786 / 1842 | 706 / 643 |
| Patients excluded | 140 / 164 | 182 / 128 |
| Patients included in ITT | 1646 / 1678 | 524 / 515 |
| Loss to follow-up | 140 / 164 patients without follow-up data were excluded in the ITT analysis, so, in the final ITT analysis, no patient lost to follow up | 11 / 13 (lost to follow-up; censored at last available follow-up) |
|  |  |  |
| Intenvention related information |  |  |
| Adherence^*^ | The extension cohort of mAFA-II primarily reflects adherence to symptom monitoring (B component); 70.8% achieved ≥70% monitoring adherence | ABC pathway adherence at 12 months: A: 85.2% vs 20.8%; B: 91.6% vs 94.6%; C: 43.1% vs 42.2% |
| Intervention Persistence | 91.7% | Design-based (continuous use) |
| Cross-over | None | None |

^*^Adherence metrics were not directly comparable across trials; mAFA-II primarily reported app-based monitoring adherence (B component), whereas MIRACLE-AF assessed adherence to each ABC pathway component. Adherence and persistence data for mAFA-II were derived from a long-term extension cohort study and were not assessed in the main trial population. Full details: Guo Y, Guo J, Shi X, et al. Mobile health technology-supported atrial fibrillation screening and integrated care: A report from the mAFA-II trial Long-term Extension Cohort. Eur J Intern Med. 2020;82:105-111. doi:10.1016/j.ejim.2020.09.024.

**Supplementary Table S2 Inclusion and exclusion criteria of the mAFA-II and MIRACLE-AF trials**

| **Trial** | **Inclusion Criteria** | **Exclusion Criteria** |
| --- | --- | --- |
| mAFA-II | (1) Age ≥18 years  (2) Diagnosed with new-onset, paroxysmal, persistent or permanent AF confirmed by ECG or 24-h Holter monitors  (3) CHA_2_DS_2_-VASc score ≥2 | (1) Age <18 years  (2) With mechanical prosthetic valve or moderate/severe mitral stenosis  (3) Inability to provide informed consent  (4) Inability to complete 1-year follow-up |
|  |  |  |
| MIRACLE-AF | (1) Age ≥65 years  (2) Diagnosed with AF  (3) Resident in rural area under study  (4) Consenting to be managed by village doctors | (1) Prior valve replacement or moderate/severe rheumatic mitral stenosis  (2) Cardiac implantable devices (e.g., ICD/CRT)  (3) AF ablation or surgery within 3 months  (4) Prior AF ablation or LAA occlusion  (5) Planned intervention  (6) Life expectancy <1 year  (7) Current enrollment in other studies |

Abbreviations: AF, atrial fibrillation; ECG, electrocardiogram; ICD, implantable cardioverter-defibrillator; CRT, cardiac resynchronisation therapy; LAA, left atrial appendage.

**Supplementary Table S3 Schoenfeld residuals test for proportional hazards assumption**

| **Study Endpoint** | **P Value for Treatment Group** | **Global P Value** |
| --- | --- | --- |
| Primary Endpoint | 0.809 | 0.130 |
| Secondary Endpoint |  |  |
| All-Cause Mortality | 0.959 | 0.218 |
| All Stroke | 0.795 | 0.982 |
| Ischaemic Stroke | 0.934 | 0.980 |
| Haemorrhage Stroke | 0.825 | 0.354 |
| Composite of HF/ACS | 0.922 | 0.089 |
| HF | 0.872 | 0.433 |
| ACS | 0.182 | 0.214 |
| Major Bleeding | 0.635 | 0.564 |

Abbreviations: ACS, acute coronary syndromeHF, heart failure; ICH, intracranial haemorrhage; IS, ischaemic stroke.

**Supplementary Table S4 Subgroup analyses of the primary endpoint**

| Subgroup | Event in Control, n (%) | Event in Intervention, n (%) | aHR (95% CI) | P Value | Interaction P Value |
| --- | --- | --- | --- | --- | --- |
| Age |  |  |  |  |  |
| Age <65 years | 26/499 (5.2) | 17/655 (2.6%) | 0.69 (0.37–1.30) | 0.254 | 0.896 |
| Age ≥65 years | 133/1694 (7.9%) | 83/1515 (5.5%) | 0.72 (0.56–0.94) | 0.016 |  |
|  |  |  |  |  |  |
| Sex |  |  |  |  |  |
| Male | 48/858 (5.6%) | 51/864 (5.9%) | 0.54 (0.39–0.74) | < 0.001 | 0.007 |
| Female | 111/1335 (8.3%) | 49/1306 (3.8%) | 1.13 (0.75–1.72) | 0.553 |  |
|  |  |  |  |  |  |
| AF Type |  |  |  |  |  |
| Non-Paroxysmal AF | 119/1493 (8%) | 70/1460 (4.8%) | 0.65 (0.49–0.86) | 0.002 | 0.109 |
| Paroxysmal AF | 40/700 (5.7%) | 30/710 (4.2%) | 0.97 (0.62–1.51) | 0.893 |  |
|  |  |  |  |  |  |
| CHA_2_DS_2_-VASc score^a^ |  |  |  |  |  |
| Low risk | 66/1145 (5.8%) | 19/1128 (1.7%) | 0.36 (0.23–0.57) | < 0.001 | < 0.001 |
| High risk | 93/1048 (8.9%) | 81/1042 (7.8%) | 0.95 (0.70–1.27) | 0.710 |  |
|  |  |  |  |  |  |
| Hypertension |  |  |  |  |  |
| No | 57/787 (7.2%) | 11/816 (1.3%) | 0.25 (0.13–0.48) | < 0.001 | < 0.001 |
| Yes | 102/1406 (7.3%) | 89/1354 (6.6%) | 0.95 (0.71–1.27) | 0.742 |  |
|  |  |  |  |  |  |
| Coronary Artery Disease |  |  |  |  |  |
| No | 123/1459 (8.4%) | 58/1524 (3.8%) | 0.52 (0.39–0.70) | < 0.001 | < 0.001 |
| Yes | 36/734 (4.9%) | 42/646 (6.5%) | 1.53 (0.92–2.53) | 0.101 |  |
|  |  |  |  |  |  |
| Heart Failure |  |  |  |  |  |
| No | 131/1805 (7.3%) | 62/1783 (3.5%) | 0.55 (0.42–0.73) | < 0.001 | < 0.001 |
| Yes | 28/388 (7.2%) | 38/387 (9.8%) | 1.46 (0.85–2.49) | 0.166 |  |
|  |  |  |  |  |  |
| Diabetes Mellitus |  |  |  |  |  |
| No | 117/1726 (6.8%) | 64/1697 (3.8%) | 0.62 (0.45–0.85) | 0.003 | 0.045 |
| Yes | 42/467 (9%) | 36/473 (7.6%) | 1.03 (0.69–1.52) | 0.898 |  |
|  |  |  |  |  |  |
| Prior Ischaemic Stroke |  |  |  |  |  |
| No | 131/1888 (6.9%) | 69/1893 (3.6%) | 0.61 (0.45–0.82) | 0.001 | 0.020 |
| Yes | 28/305 (9.2%) | 31/277 (11.2%) | 1.22 (0.74–2.00) | 0.435 |  |
|  |  |  |  |  |  |
| Chronic Kidney Disease |  |  |  |  |  |
| No | 117/1926 (6.1%) | 72/1949 (3.7%) | 0.70 (0.54–0.92) | 0.010 | 0.728 |
| Yes | 42/267 (15.7%) | 28/221 (12.7%) | 0.77 (0.47–1.28) | 0.320 |  |
|  |  |  |  |  |  |
| Baseline Anticoagulants Use |  |  |  |  |  |
| No | 115/1338 (8.6%) | 46/1027 (4.5%) | 0.49 (0.35–0.70) | < 0.001 | 0.001 |
| Yes | 44/855 (5.1%) | 54/1143 (4.7%) | 1.24 (0.82–1.88) | 0.302 |  |
|  |  |  |  |  |  |
| Baseline Statins Use |  |  |  |  |  |
| No | 125/1586 (7.9%) | 60/1475 (4.1%) | 0.59 (0.44–0.78) | < 0.001 | 0.008 |
| Yes | 34/607 (5.6%) | 40/695 (5.8%) | 1.18 (0.74–1.89) | 0.481 |  |

^a^For CHA₂DS₂-VASc risk, males ≥3 or females ≥4 is considered high risk.

Hazard ratios (HRs) and 95% confidence intervals (CIs) were estimated using trial-stratified Cox proportional hazards models with robust standard errors clustered at the site level. Models were adjusted for the CHA₂DS₂-VASc score, HAS-BLED score, age, comorbidities (chronic kidney disease, coronary artery disease, liver dysfunction), and baseline medications (warfarin, dabigatran, aspirin, amiodarone, beta-blockers, diuretics, and gastric antiacid drugs). Subgroup analyses were performed by including interaction terms between treatment and subgroup variables. P values for interaction are reported. Subgroup analyses should be interpreted as exploratory.

Abbreviations: AF, atrial fibrillation; aHR, adjusted hazard ratio; CI, confidence interval.

**Supplementary Table S5 Sensitivity analyses of primary endpoint using stepwise covariate adjusted mixed-effects Cox models**

| **Model specification** | **aHR (95% CI)** | **P Value** |
| --- | --- | --- |
| Mixed-effects Cox model with site-level random intercept | 0.72 (0.54–0.96) | 0.018 |

Adjusted hazard ratios (aHRs) were estimated using mixed-effects Cox proportional hazards models with a site-level random intercept to account for within-cluster correlation. Models were sequentially adjusted for CHA₂DS₂-VASc score, HAS-BLED score, age, comorbidities (chronic kidney disease, coronary artery disease, liver dysfunction), and baseline medications (warfarin, dabigatran, aspirin, amiodarone, beta-blockers, diuretics, and gastric antiacid drugs).

Abbreviations: aHR, adjusted hazard ratio; CI, confidence interval.

**Supplementary Table S6 Sensitivity analyses of non-fatal secondary endpoints using Fine-Gray subdistribution hazard models accounting for competing risk of death**

| **Non-Fatal Secondary Endpoint** | **SHR (95% CI)** | **P Value** |
| --- | --- | --- |
| All Stroke | 0.78 (0.38–1.62) | 0.509 |
| IS | 0.88 (0.36–2.10) | 0.766 |
| ICH | 0.67 (0.19–2.33) | 0.525 |
| Composite of HF/ACS | 0.62 (0.40–0.95) | 0.029 |
| HF | 0.73 (0.47–1.14) | 0.163 |
| ACS | 0.24 (0.06–0.88) | 0.031 |
| Major Bleeding | 1.11 (0.69–1.79) | 0.653 |

SHRs and 95% CIs were estimated using Fine–Gray competing risk models adjusted for CHA₂DS₂-VASc score, HAS-BLED score, age, comorbidities (chronic kidney disease, coronary artery disease, liver dysfunction), and baseline medications (warfarin, dabigatran, aspirin, amiodarone, beta-blockers, diuretics, and gastric antiacid drugs), with all-cause death treated as a competing event.

Abbreviations: ACS, acute coronary syndrome; CI, confidence interval; HF, heart failure; ICH, intracranial haemorrhage; IS, ischaemic stroke; SHR, subdistribution hazard ratio.

**Supplementary Table S7 Sensitivity analyses of primary endpoint using truncated follow-up durations (6 and 12 months)**

| **Follow Up Period** | **Events in Intervention, n (%)** | **Events in Control, n (%)** | **aHR (95% CI)** | **P Value** |
| --- | --- | --- | --- | --- |
| 6 Months | 50 (2.3) | 85 (3.9) | 0.71 (0.49–1.03) | 0.068 |
| 12 Months | 91 (4.2) | 145 (6.6) | 0.73 (0.55–0.96) | 0.027 |

HRs and 95% CIs were estimated using Cox proportional hazards models with follow-up truncated at prespecified time points, adjusted for CHA₂DS₂-VASc score, HAS-BLED score, age, comorbidities (chronic kidney disease, coronary artery disease, liver dysfunction), and baseline medications (warfarin, dabigatran, aspirin, amiodarone, beta-blockers, diuretics, and gastric antiacid drugs), and trial (stratified), with clustering accounted for at the site level. Events are presented as number of events over total participants in each group.

Abbreviations: aHR, adjusted hazard ratio; CI, confidence interval.

**Supplementary Table S8 Sensitivity analyses of primary endpoint using landmark analyses at 1 and 3 months**

| **Landmark Time** | **Events in Intervention, n (%)** | **Events in Control, n (%)** | **aHR (95% CI)** | **P Value** |
| --- | --- | --- | --- | --- |
| 1 Month | 94/2051 (4.6) | 145/2167 (6.7) | 0.74 (0.57–0.95) | 0.021 |
| 3 Months | 76/1820 (4.2) | 124/2025 (6.1) | 0.69 (0.52–0.92) | 0.011 |

HRs and 95% CIs were estimated using Cox proportional hazards models with follow-up starting from prespecified landmark time points, adjusted for CHA₂DS₂-VASc score, HAS-BLED score, age, comorbidities (chronic kidney disease, coronary artery disease, liver dysfunction), and baseline medications (warfarin, dabigatran, aspirin, amiodarone, beta-blockers, diuretics, and gastric antiacid drugs), stratified by trial and accounting for clustering at the site level. Events are presented as number of events over total participants in each group.

Abbreviations: aHR, adjusted hazard ratio; CI, confidence interval.

**Supplementary Table S9. Analyses of original trial-specific primary endpoints within each trial population and comparison with the harmonised composite endpoint used in the pooled analysis**

|  | Population | Endpoint | aHR (95% CI) | P |
| --- | --- | --- | --- | --- |
| Harmonised endpoint analysis | Pooled | Harmonised endpoint^a^ | 0.72 (0.56–0.93) | 0.011 |
| Trial-specific endpoint analysis | mAFA-II | Original primary endpoint^b^ | 0.38 (0.22–0.65) | < 0.001 |
|  | MIRACLE-AF | Original primary endpoint^c^ | 0.45 (0.29–0.71) | < 0.001 |

^a^Harmonised composite endpoint defined as all-cause mortality, ischaemic stroke, intracranial haemorrhage, heart failure, acute coronary syndrome, and major bleeding.

^b^Original primary endpoint in the mAFA-II trial: composite of ischaemic stroke/systemic thromboembolism, all-cause mortality, and rehospitalisation.

^c^Original primary endpoint in the MIRACLE-AF trial: composite of cardiovascular death, stroke, hospitalisation for heart failure or acute coronary syndrome, and AF-related emergency visits.

Adjusted hazard ratios (aHRs) were estimated using Cox proportional hazards models within each trial population.

Abbreviations: aHR, adjusted hazard ratio; CI, confidence interval.
